# Supplementary material for: Lidocaine Enhanced Antitumor Efficacy and Relieved Chemotherapy-Induced Hyperalgesia in Mice with Metastatic Gastric Cancer
Source: Int J Mol Sci. 2025 Jan 19;26(2):828. doi: 10.3390/ijms26020828 (PMC11766172; doi:10.3390/ijms26020828)
Supplement: Supplementary file 1 [file ijms-26-00828-s001.zip › ijms-3419952-supplementary.pdf]

Lidocaine enhanced antitumor efficacy and relieved chemotherapy-induced hyperalgesia in mice with metastatic gastric cancer

Supplementary data

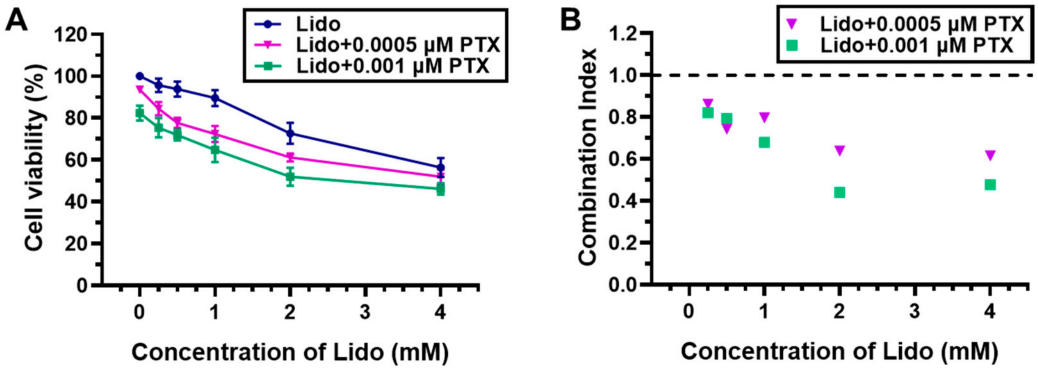

Figure S1. Synergistic cytotoxicity of Lido and PTX on human gastric cancer cell HGC-27. (A) Viability of cells treated with Lido and PTX at different concentrations (n=3). (B) CI values of Lido and PTX at different concentrations.

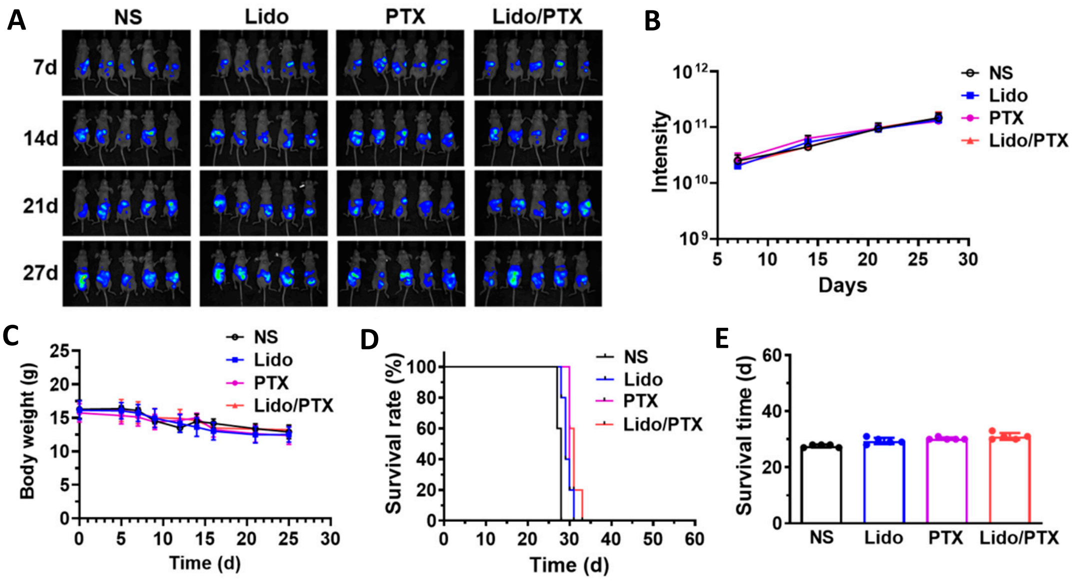

Figure S2. Small-volume (200 μL) intraperitoneal chemotherapy for peritoneal metastasis model of gastric cancer. (A) Luminescence images obtained through IVIS imaging weekly. (B) Quantification of luminescence measurements. (C) body weight, (D) Survival rate and (E) survival time of mice.

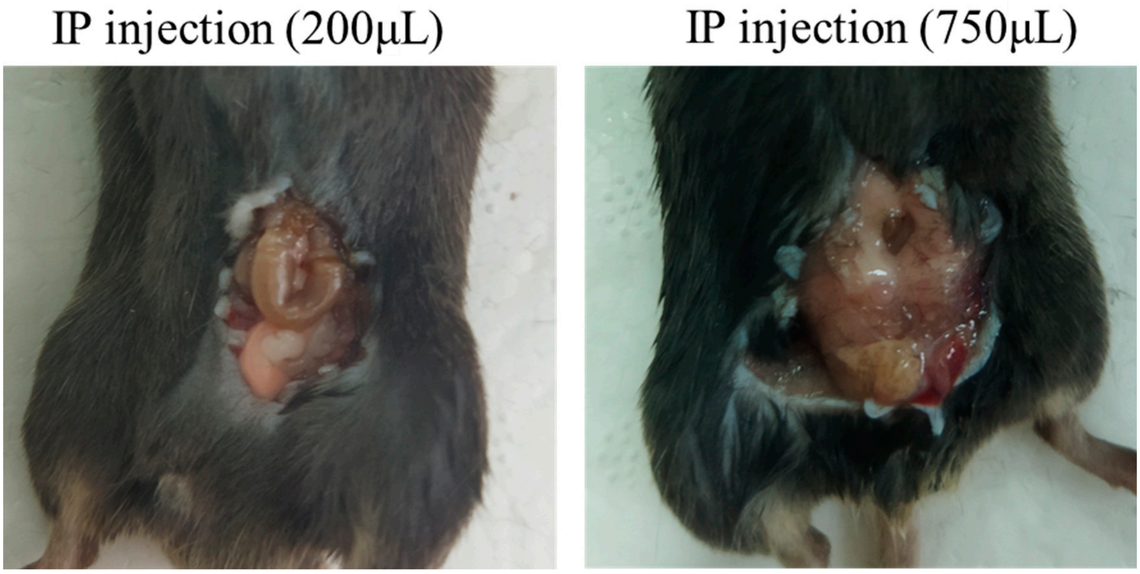

Figure S3. Retention of drugs in the peritoneal cavity 1 hour after injection with volume of 200 μL or 750 μL.
